# In Vitro Study of the Metabolic Characteristics of Eight Isoquinoline Alkaloids from Natural Plants in Rat Gut Microbiota

**DOI:** 10.3390/molecules22060932

**Published:** 2017-06-04

**Authors:** Chi-Yu He, Jie Fu, Jia-Wen Shou, Zhen-Xiong Zhao, Long Ren, Yan Wang, Jian-Dong Jiang

**Affiliations:** State Key Laboratory of Bioactive Substance and Function of Natural Medicines, Institute of Materia Medica, Chinese Academy of Medical Sciences/Peking Union Medical College, Beijing 100050, China; hechiyu1028@163.com (C.-Y.H.); fujie@imm.ac.cn (J.F.); shoujiawen@163.com (J.-W.S.); zhaozhenxiong@imm.ac.cn (Z.-X.Z.); renlong36@imm.ac.cn (L.R.)

**Keywords:** isoquinoline alkaloids, gut microbiota, metabolites, docking, 14α-demethylase, LC/MS^n^-IT-TOF

## Abstract

Gut microbiota is populated with an immense number of microorganisms, which can be regulated by dietary components and drugs to markedly affect the nutritional and health status of the host. Eight medicinal isoquinoline alkaloids from natural plants were cultured anaerobically with rat gut microbiota and an LC/MS^n^-IT-TOF technique was used to identify the resulting metabolites. Palmatine, tetrahydropalmatine, dauricine, and tetrandrine containing nitro-hexatomic isoquinoline rings could be easily transformed by the intestinal flora in vitro and a total of nine demethylated metabolites were detected. However, sinomenine, homoharringtonine, harringtonine, and galanthamine, which all contained benzazepine, could not undergo demethylation. Computer-assisted docking was used to analyze the binding between these compounds and sterol 14α-demethylase. The computational results demonstrated that hydrophobic interactions were the main driving force for binding, but the steric hindrance produced by the benzazepine structure resulted in a weak interaction between the hit compounds and the enzyme. This work illustrated that gut microbiota were important in the metabolism of isoquinoline alkaloids.

## 1. Introduction

Human gut microbiota represents a complex community of vast and diverse microorganisms that reside in the digestive system and contain a hundred times more genes than our own human genome [1]. Referred to as a “hidden organ” of the body, the microorganisms are considered to be an important internal environmental factor related to human health and disease; a close association with many health problems has been reported, including obesity, metabolic syndrome, cardiovascular diseases, and diabetes [2,3,4]. A variety of enzymes can be found in the intestinal flora, including β-glucuronidase, β-glucosidase, β-galactase, nitroreductase, azoreductase, 7α-hydroxylase, protease, and various carbohydrates [5]. These enzymes can interact with drugs and generate many metabolites that differ from those that are generated by other host organs [6,7], which indicates that the intestinal flora has a good metabolic capacity. This has been a well-studied topic in the field of drug metabolism research, especially with regard to orally administered drugs, as the metabolic reactions by gut microbiota might alter the chemical structure and biological effects of drugs. The metabolites generated by gut microbiota might increase toxicity, show new bioactivities, or promote intestinal absorption by regulation of the gut microbiota [8,9]. Thus, drug metabolism in the intestinal bacterial is an attractive and indispensable component of pharmaceutical research and drug investigation. In this study, we present eight medicinal isoquinoline alkaloids, palmatine, tetrahydropalmatine, dauricine, tetrandrine, sinomenine, homoharringtonine, harringtonine, and galanthamine, as research topic to demonstrate the significance of drug metabolism by gut microbiota.

The eight isoquinoline alkaloids have a wide range of pharmacological activity. For example, tetrahydropalmatine and sinomenine show antinociceptive effects and may be a useful analgesic in the management of neuropathic pain [10,11]; dauricine exhibits anti-inflammatory effects [12]; tetrandrine has both anti-inflammatory and anti-arrhythmia effects [13,14]; homoharringtonine and harringtonine have been demonstrated as potential leukemia and cancer treatments [15,16]; evidence also showed that galanthamine improved cognitive and behavioral functions in Alzheimer’s disease [17]. However, as these isoquinoline alkaloids exhibit low oral bioavailability, their absorption into the bloodstream is relatively difficult. Previous pharmacokinetics studies have reported that 6.59% of an oral dose of tetrahydropalmatine was absorbed [18], the absolute bioavailability of dauricine in rat was 16.6% and palmatine showed poor permeability and absorption [19,20]. These findings mentioned above showed that the isoquinoline alkaloids might have good efficacy despite their poor bioavailability, which attracted us to explain the interesting question. Our present study revealed that gut microbiota will be an important factor for an improved understanding of metabolic mechanism for these isoquinoline alkaloids in vivo. 

In this study, eight isoquinoline alkaloids (either with a hexatomic ring or with a heptatomic ring) were incubated anaerobically with rat intestinal bacteria. HPLC coupled with electrospray ionization (ESI)—ion trap-time of flight mass spectrometry (LC/MS^n^-IT-TOF) was employed to identify and characterize the metabolites. Based on the results, we obtained preliminary knowledge of a possible metabolic pathway for these isoquinoline alkaloids in intestinal flora in vitro; subsequently, we performed a computer-assisted docking analysis of the initial step of the metabolism reaction catalyzed by the sterol 14α-demethylase and indicated that the biotransformation of the isoquinoline alkaloids potentially related to the chemical structure with hexatomic ring of the parent drugs. The results will therefore provide partial evidence to further explore the clinical efficacy of drugs from the aspect of gut microbiota.

## 2. Results and Discussion

**Figure 1:**
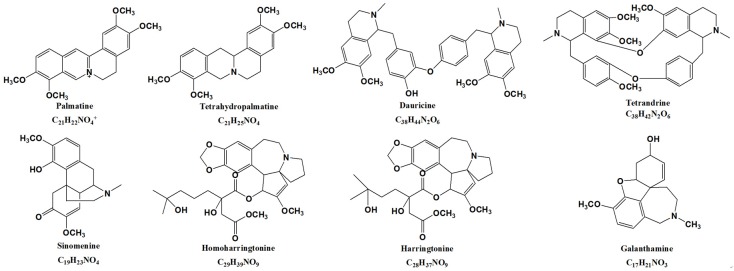
The chemical structure of eight isoquinoline alkaloids: palmatine, tetrahydropalmatine, dauricine, tetrandrine, sinomenine, homoharringtonine, harringtonine, and galanthamine.

To investigate the metabolism of isoquinoline alkaloids (Figure 1) (Palmatine, tetrahydropalmatine, dauricine, tetrandrine, sinomenine, homoharringtonine, harringtonine, and galanthamine) in gut microbiota, we incubated all the compounds with colonic material. Briefly, colon contents from six rats were pooled, and the sample was transferred into a flask containing the anaerobic medium. After thorough mixing, the cultures (which contained intestinal bacteria and anaerobic medium) were pre-incubated under anaerobic conditions with a N_2_ atmosphere at 37 °C for 60 min. Each compound was added into the fresh rat intestinal bacteria cultures. The cultures were incubated at 37 °C for 48 h in the presence or absence of rat intestinal bacteria. After the reactions were terminated and each sample was prepared, LC/MS^n^-IT-TOF was used for online analysis of their metabolites. The results showed that palmatine, tetrahydropalmatine, dauricine, and tetrandrine were transformed by the intestinal flora in vitro. In contrast, no metabolites of the other four isoquinoline alkaloids in intestinal microflora were detected in this study. 

### 2.1. Identification of Palmatine Metabolites in Rat Intestinal Bacteria

**Figure 2:**
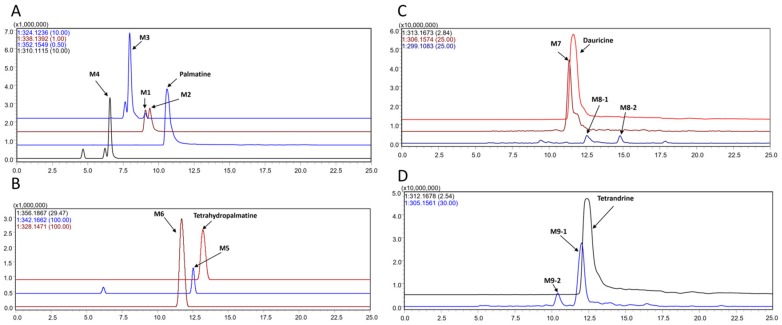
Extracted ion chromatograms (EICs) of metabolites in the incubation of palmatine (**A**), tetrahydropalmatine (**B**), dauricine (**C**), and tetrandrine (**D**) in the intestinal bacteria in vitro (positive) ion mode, the *m*/*z* values in this chromatogram: A, palmatine, 352.1549; M1, 338.1392; M2, 338.1392; M3, 324.1236; M4, 310.1115. B: tetrahydropalmatine, 326.1867; M5, 342.1662; M6, 328.1471. C: dauricine, 313.1673; M7, 306.1574; M8, 299.1083. D: tetrandrine, 312.1678; M9, 305.1561.

Four metabolites, together with palmatine, were detected by comparison with a blank sample (Figure 2A). The metabolites were marked as M1–M4 according to their HPLC retention times and extracted ion chromatograms (EICs). The metabolites of palmatine (M1-M4) showed good responses in positive ion mode.

The [M + H]^+^ of the parent drug (t_R_ = 10.9 min) was at *m*/*z* 352.1549; the mass spectral data of the parent drug and metabolites are listed in Table 1.

M1 (t_R_ = 9.2 min) and M2 (t_R_ = 9.5 min) both showed an [M + H]^+^ peak at *m*/*z* 338.1932; the two metabolites are isomers. With regard to the molecular ion in positive mode ([M + H]^+^), the *m*/*z* value of M1 and M2 was 14 Da lower than that of the parent drug. These results demonstrated that M1 and M2 were formed via the loss of a CH_2_ group from the methoxyl group of palmatine. Reference samples (columbamine and jatrorrhizine) were used to identify the structures of M1 and M2, respectively. After comparing the retention time and fragmentation patterns with standard substances, M1 was generated via the loss of a CH_2_ group from the methoxyl group at the C-2 position and was identified as 5, 6-dihydro-2-hydroxy-3, 10, 11-trimethoxydibenzo[a,g]quinolizinium, also known as columbamine; while M2 was formed via the loss of a CH_2_ group from the methoxyl group at the C-3 position and identified as 3-hydroxy-2, 9, 10-trimethoxy-5,6-dihydroisoquino[3, 2-a] isoquinolinium, also known as jatrorrhizine. 

M3 (t_R_ = 8.2 min) had an [M + H]^+^ at *m*/*z* 324.1236. As the *m*/*z* value of M3 was 28 Da lower than that of the parent drug and 14 Da lower than that of M1 and M2, we proposed that M3 might be transformed from M1 and M2 via a demethylation reaction. After comparison with the reference subject, M3 was inferred to be demethyleneberberine (dibenzo [a, g] quinolizinium, 5, 6-dihydro-2, 3-dihydroxy-9, 10-dimethoxychloride). 

M4 (t_R_ = 6.6 min) had an [M + H]^+^ at *m*/*z* 310.1115, which was 42 Da lower than that of the parent drug and 14 Da lower than that of M3, thus it was suggested that M4 might be generated from M3 after a loss of CH_2_. It was considered to be the demethylation compound of demethyleneberberine (M3).

**Figure 3:**
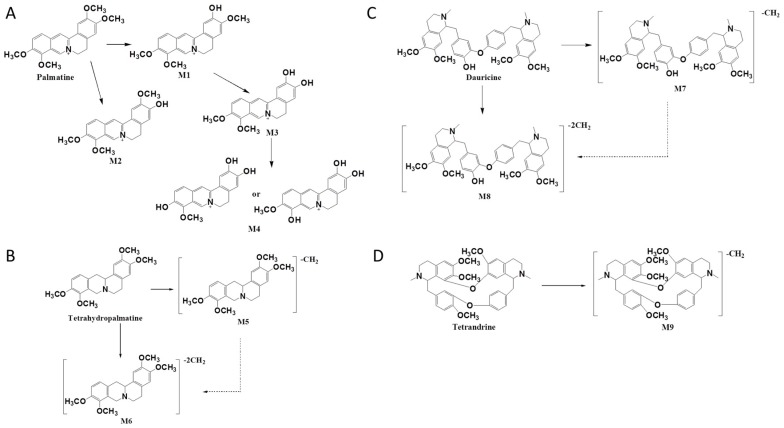
The proposed metabolic pathways of palmatine (**A**), tetrahydropalmatine (**B**), dauricine (**C**), and tetrandrine (**D**) in intestinal flora after incubation in vitro.

In summary, four metabolites of palmatine were detected in the rat intestinal bacteria incubation system: columbamine (M1), jatrorrhizine (M2), demethyleneberberine (M3), and the demethylation compound of demethyleneberberine (M4). The proposed metabolic pathway of palmatine is shown in Figure 3A.

### 2.2. Identification of Tetrahydropalmatine Metabolites in Rat Intestinal Bacteria

Two metabolites, together with tetrahydropalmatine, were detected by comparison with the blank sample (Figure 2B). The metabolites were marked as M5–M6 in accordance with their HPLC retention times and EICs. The metabolites of tetrahydropalmatine (M5–M6) showed good response in the positive ion mode.

The parent drug (t_R_ = 13.5 min) had an [M + H]^+^ at *m*/*z* 356.1867. The mass spectral data of the parent drug and metabolites are listed in Table 1.

M5 (t_R_ = 12.6 min) had an [M + H]^+^ at *m*/*z* 342.1662; based on the *m*/*z* value of M5, which had a loss of a CH_2_ from tetrahydropalmatine, it was obtained from demethylation of the parent. 

M6 (t_R_ = 11.8 min) had an [M + H]^+^ at *m*/*z* 328.1471. The *m*/*z* value of M6 was 14 Da lower than that of M5 and 28 Da lower than that of the parent drug, which indicated that M6 was formed by loss of two CH_2_ groups from the parent drug or a CH_2_ group from M5 via a demethylation reaction.

In summary, the two metabolites of tetrahydropalmatine that were detected in the rat intestinal bacteria incubation system were generated by the loss of one or two CH_2_ groups through demethylation reactions. The proposed metabolic pathway of tetrahydropalmatine is shown in Figure 3B.

### 2.3. Identification of Dauricine Metabolites in Rat Intestinal Bacteria

Three metabolites, together with dauricine, were detected by comparison with the blank sample (Figure 2C). The metabolites were marked as M7–M8 according to their HPLC retention times and EICs. The metabolites of dauricine (M7–M8) showed good response in positive ion mode. 

Owing to the presence of two nitrogen atoms in the structure of dauricine, the parent drug and metabolites more easily formed a double-charged ion in positive ion mode; therefore, the parent drug (t_R_ = 11.9 min) had an [M + 2H]^2+^ at *m*/*z* 313.1673 (2). The MS^2^ spectrum of dauricine had fragments at *m*/*z* 297.6498 (2) (M − 32 Da) and MS^3^ spectrum had fragments at *m*/*z* 290.6039 (2) (M − 32 Da − 14 Da), *m*/*z* 282.1284 (2) (M − 32 Da − 14 Da − 16 Da).

M7 (t_R_ = 11.4 min) showed a quasi-molecular ion of [M + 2H]^2+^ at *m*/*z* 306.1574 (2), and the MS^2^ spectrum had fragments at *m*/*z* 290.6376 (2) (M − 32 Da), *m*/*z* 282.1292 (2) (M − 32 Da − 16Da). When the *m*/*z* 306.1574 (2) ion was further fragmented in the MS^3^ spectrum, an ion at *m*/*z* 275.0978 was produced. Compared with the fragmentations of the parent drug (*m*/*z* 313.1673 (2)), it was suggested that the *m*/*z* value of M7 (*m*/*z* 306.1574 (2)) was 14 Da lower, which indicated that M7 was formed by the loss of a CH_2_ group from the parent drug.

The isomers M8-1 (t_R_ = 12.6 min) and M8-2 (t_R_ = 14.8 min) both had [M + 2H]^2+^ peaks at *m*/*z* 299.1083 (2). The MS^2^ spectrum had fragments at *m*/*z* 283.6370 (M − 32 Da,) and the MS^3^ spectrum had fragments at *m*/*z* 268.1075 (M − 32 Da − 14 Da − 16 Da). A comparison of the fragmentation data for M8 and M7 revealed two important observations. First, the *m*/*z* value of M8 was 14 Da lower than that of M7, which suggested that M8 lost a CH_2_ group via a demethylation reaction; second, the fragmentations of M8 and M7 were very similar to each other. For example, a comparison of the fragments at *m*/*z* 283.6370 and 268.1075 of M8 with those at *m*/*z* 290.6376 and 275.0978 of M7, which were only 7 Da different, demonstrated that the fragmentation pathways of the two metabolites were similar. Therefore, M8 was obtained by the loss of two CH_2_ groups from the parent drug or a CH_2_ group from M7 via a demethylation reaction.

In summary, three metabolites of dauricine were detected in the rat intestinal bacteria incubation system and were transformed from demethylation reactions by the loss of one or two CH_2_ groups. The proposed metabolic pathway of dauricine is shown in Figure 3C and the mass spectral data of dauricine and its metabolites are listed in Table 1.

### 2.4. Identification of Tetrandrine Metabolites in Rat Intestinal Bacteria

Similar to dauricine, tetrandrine, which eluted at 12.9 min contained two nitrogen atoms and formed a double-charged ion in positive ion mode, had an [M + 2H]^2+^ at *m*/*z* 312.1678 (2).

Two metabolites, together with the tetrandrine, were detected by comparison with the blank sample (Figure 2D). The metabolites were marked as M9 according to its HPLC retention times and extracted ion chromatograms (EICs). The metabolites of dauricine (M9) showed good responses in positive ion mode. 

M9-1 (t_R_ = 10.5 min) and M9-2 (t_R_ = 12.0 min) were isomers; both had a quasi-molecular ion of [M + 2H]^2+^ at *m*/*z* 305.1561 (2). Their molecular weights were 14 Da less than that of tetrandrine, which suggested that they were obtained by the loss of one CH_2_ group from tetrandrine. 

In summary, the two metabolites of tetrahydropalmatine that were detected in the rat intestinal bacteria incubation system were generated by the loss of one CH_2_ group through the demethylation reaction. The proposed metabolic pathway of palmatine is shown in Figure 3D and the mass spectral data of the parent drug and metabolites are listed in Table 1.

### 2.5. Molecular Docking Between Isoquinoline Alkaloids and Sterol 14α-Demethylase

**Figure 4:**
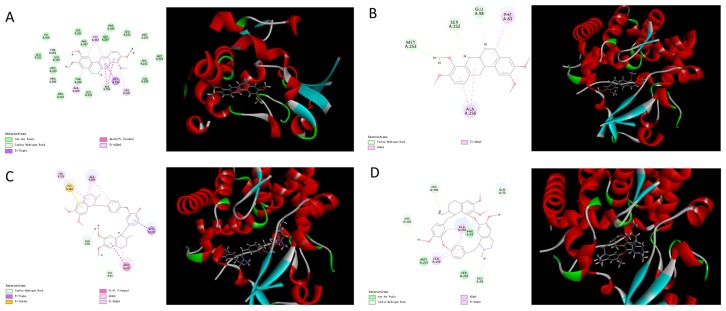
Molecular docking between isoquinoline alkaloids [(**A**) palmatine; (**B**) tetrahydropalmatine; (**C**) dauricine; (**D**) tetrandrine] and sterol 14α-demethylase.

**Figure 5:**
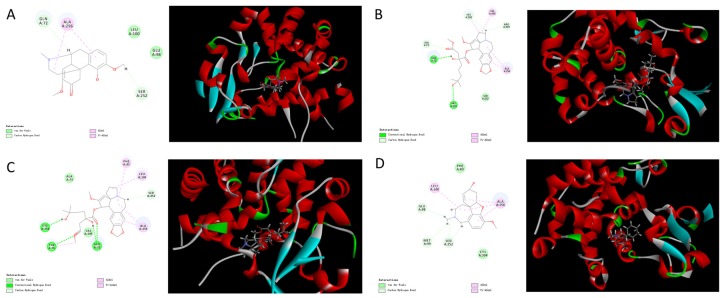
Molecular docking between isoquinoline alkaloids [(**A**) sinomenine; (**B**) homoharringtonine; (**C**) harringtonine; (**D**) galanthamine] with sterol 14α-demethylase.

Sterol 14α-demethylase (CYP51) is a cytochrome P450 heme thiolate-containing enzyme involved in the biosynthesis of membrane sterols in all biological kingdoms from bacteria to animals [21]. Since the crystal structures of CYP51 have been illustrated and are available in the Protein Data Bank (www.rcsb.org, PDB, IEIE) [22], we performed a computer-assistant docking analysis of the initial binding step, using the Discovery Studio Client software (v16.1.0.15350). The putative chemical mechanism for the docking of eight isoquinoline alkaloids by CYP51 is shown in Figure 4 and Figure 5.

Based on the results of molecular docking, we found four compounds that contained nitro-hexatomic isoquinoline rings (palmatine, tetrahydropalmatine, dauricine, and tetrandrine) showed a good docking score and interaction with active site amino acids. They exhibited excellent docking performance when docking onto CYP51 with a binding free energy of −18.99 kcal/mol, −5.82 kcal/mol, −3.81 kcal/mol, and −21.22 kcal/mol, respectively. The analysis of the binding mode of these compounds revealed the interactions with the conserved active side residues of CYP51. The results demonstrated that the presence of hydrophobic contacts with a considerable number of residues in the active site appeared to be pivotal in compound binding and these residues contributed to the substrate specificity of CYP51 from various species. For example, palmatine showed van der Waals’ interaction with Gly257 and π-σ interaction with Gly396; tetrahydropalmatine exhibited π-alkyl interaction with Ala256 and Phe83; dauricine showed π-π interaction with Phe83; and tetrandrine exhibited van der Waals’ interaction with Phe83, Ser252, and Met253. Therefore, three out of four hit compounds were involved in interactions with Phe83, which is the active-site residue in CYP51 family.

However, the other four isoquinoline alkaloids that contained benzazepine (sinomenine, homoharringtonine, harringtonine, and galanthamine) showed a poor performance when docking onto CYP51 with binding free energies of 9.13 kcal/mol, 12.38 kcal/mol, 21.75 kcal/mol, and 17.31 kcal/mol, respectively. Although the compounds can form hydrogen bonds with CYP51 (homoharringtonine formed conventional hydrogen bonds with Gln72 and His259; harringtonine formed conventional hydrogen bonds with Cys394, Tyr76, and Gln72), the presence of the benzazepine structure produced a steric hindrance effect. The twisted structure resulted in a weak interaction between the hit compounds and CYP51, which led to a poor combination between them.

In summary, palmatine, tetrahydropalmatine, dauricine, and tetrandrine established good binding combinations with CYP51 and the results indicated that the hydrophobic interactions were very important for binding interactions between the ligands and the active site residue. Additionally, compounds that contain a benzazepine structure, such as sinomenine, homoharringtonine, harringtonine, and galanthamine, generally produce the steric hindrance effect. The twisted structure is a larger-sized group able to form a strong resistance, which resulted in a weak combination between the hit compounds and the protein, which affected the chemical interactions. Thus, the computational results were validated by the in vitro experiments (palmatine, tetrahydropalmatine, dauricine, and tetrandrine could be easily transformed to demethylated metabolites, but the other four isoquinoline alkaloids could not undergo the biotransformation), suggesting that demethylase CYP51 might be an important bacterial enzyme in these reactions and CYP51 or other metabolic enzymes with similar functions might exist in the gut microbiota. This explains that the concept of microbiota as a metabolic “organ” that contains many enzymes and exerts a powerful metabolic capacity. Therefore, it’s critical necessary to conduct extensive in vivo studies to validate the results and find enzymes like CYP51 or other enzymes with similar functions on the intestinal bacterial communities. Taking advantage of molecular biology techniques, these results will provide valuable points to explain the binding sites between hit compounds and enzymes and help us to find the metabolic pathway of isoquinoline alkaloids on gut microbiota. Additionally, metabolism by the intestinal microbiota could alter drug absorption and efficacy. For example, in our previous study, we observed that the gut microbiota converted berberine (BBR), a medicinal isoquinoline alkaloid isolated from *Coptis chinensis*, into dihydroberberine (dhBBR) in the intestine via a reduction reaction mediated through bacterial nitroreductase [23]. The gut microbiota converts BBR into the intestine-absorbable form of dhBBR, which has an intestinal absorption rate 5-fold higher than that of BBR in animals. Oral administration of antibiotics decreased the number of intestinal bacteria and thus suppressed the BBR to dhBBR conversion, which accordingly decreased the absorption of BBR and its lipid- and glucose-lowering efficacy. These findings, regarding the bacterial-caused structural modification of BBR in the intestine, as well as the changes in its absorption and therapeutic effects, led us to suggest that the investigation of drug metabolism by the gut microbiota should be an important step in drug development.

## 3. Materials and Methods

### 3.1. Materials and Reagents

Palmatine (No. MUST-15032604, purity > 98%), tetrahydropalmatine (No. MUST-14110412, purity > 98%), dauricine (No. 111867-201202, purity > 98%), tetrandrine (No. 110711-200708, purity > 98%), sinomenine (No. 110774-200507, purity > 98%), homoharringtonine (No. 111533-200403, purity > 98%), galanthamine (No. 100050-200802, purity > 98%), demethyleneberberine (No, MUST-12090201, purity > 98%) and jatrorrhizine (No. MUST 16040702, purity > 98%) were purchased from the National Institute for the Control of Pharmaceutical and Biological Products (Beijing, China). Harringtonine (No. SH8840, purity > 98%) and columbamine (No. SC9420, purity > 98%) were purchased from Beijing Solarbio Science & Technology Co., Ltd. (Beijing, China). Beef extract, peptone, and nutrient agar were supplied by Beijing Aoboxing Biotech Company Ltd. (Beijing, China). HPLC-grade acetonitrile, methanol, and other analytical grade reagents were purchased from Beijing Chemical Reagent Co., Ltd. (Beijing, China). The distilled water was Wahaha purified water.

### 3.2. Animals

Sprague-Dawley rats (male, 200–250 g) were obtained from the Experimental Animal Science Department of Peking University Health Science Center (Beijing, China) with the license No. SCXK (Beijing, China) 2014-0013 and housed in controlled conditions (12-h light/dark cycle, 8:00 a.m. to 8:00 p.m.; ambient temperature, 20–25 °C; relative humidity, 40–70%) with free access to a normal standard chow diet and water. The animals were provided with sawdust bedding material and were permitted a 1-week acclimatization period prior to the experiments. All animals were fasted for 12 h before experimental studies. The research was conducted in accordance with the institutional guidelines and ethics and approved by the Laboratories Institutional Animal Care and Use Committee of the Chinese Academy of Medical Sciences and the Peking Union Medical College.

### 3.3. Preparation of the Anaerobic Medium Broth and Intestinal Bacteria Culture Solution

The anaerobic medium broth for intestinal bacteria culture was prepared according to a previously published method [24]. Briefly, the following solutions were prepared: solution A (0.78% K_2_HPO_4_), solution B (0.47% KH_2_PO_4_, 1.18% NaCl, 1.2% (NH_4_)_2_SO_4_, 0.12% CaCl_2_, and 0.25% MgSO_4_·H_2_O), solution C (8% Na_2_CO_3_), solution D (0.5 g l-cysteine, 1 g beef extract, 1 g peptone, and 1 g nutrient agar dissolved in 20 mL distilled water), and a solution of 25% l-ascorbic acid. A mixture of 37.5 mL solution A, 37.5 mL solution B, 50 mL solution C, 20 mL solution D, and 2 mL l-ascorbic acid solution was prepared. The pH of the mixture was adjusted to approximately 7.5, the complete solution was transferred to a 1-L volumetric flask, and the final volume was adjusted to 1000 mL with distilled water. The obtained anaerobic medium was autoclaved at 0.1 MPa and 121 °C for 20 min in tubes, which were used immediately after cool-down or stored at 4 °C for up to one month. 

The intestinal bacteria culture solution was prepared according to a previously published method [25]. The rats were sacrificed by cervical dislocation under anesthesia, the abdomen was incised, and colonic material was immediately extracted in an anaerobic chamber. The fresh contents were homogenized under anaerobic conditions and 1 g of fecal homogenate was transferred to a flask containing 20 mL anaerobic medium, which was sparged with N_2_. After mixing, the cultures were incubated under anaerobic conditions (N_2_ atmosphere) at 37 °C for 60 min and the culture solution containing intestinal bacteria was collected.

### 3.4. Sample Preparation

The sample preparation of the eight isoquinoline alkaloids for the in vitro metabolism experiment was as follows: Two milligrams of each compound (palmatine, tetrahydropalmatine, dauricine, tetrandrine, sinomenine, homoharringtonine, harringtonine, and galanthamine) was weighed and dissolved in 1 mL methanol. A 10 μL aliquot of each solution was added to an individual sample of fresh intestinal bacteria solution (1 mL); methanol (10 μL) was used as the negative control. The cultures were incubated at 37 °C for 0, 6, 12, 18, 24, 36, and 48 h. After the reactions were terminated with acetonitrile (1 mL), the samples were vortex-mixed for 30 s and then centrifuged at 10,000× *g* for 15 min. The supernatant was dried under N_2_ flow at room temperature (20 °C–25 °C) and the resulting residue was dissolved in 200 μL methanol and filtered through a 0.22-μm micropore membrane. For each culture solution, 10 μL was analyzed by LC/MS^n^-IT-TOF. 

### 3.5. Instruments and Analytical Conditions

An HPLC-electrospray ionization-ion trap-time of fight mass spectrometry (Shimadzu, Kyoto, Japan), the coupling of ion trap (IT) having multi-stage mass analytical capacity with time of fight (TOF) mass spectrometry, was applied for on-line analysis of the metabolites of isoquinoline alkaloids in our study. The liquid chromatography system was equipped with a DGU-20A3 degasser, an LC-20AD binary pump, an SIL-20AC auto sampler, a CTO-20A column oven, and an SPD-M20A diode array detector. An Alltima C_18_ column (150 × 4.6 mm, 5 μM) was used for separation. 

For the analysis of palmatine (Table 2), the mobile phase consisted of (A) 0.5% formic acid in water and (B) acetonitrile and a gradient elution was employed for separation. The mobile phase gradient was programmed as follows: 15–45% eluent B from 0 to 10 min, 45–60% eluent B from 10 to 15 min, 60–80% eluent B from 15 to 18 min, and 80–15% eluent B from 18 to 25 min. The flow rate was 0.8 mL·min^−1^ and the detection wavelength and the column temperature were set to 347 nm and 40 °C, respectively. 

For the analysis of sinomenine (Table 2), the mobile phase consisted of (A) 0.1% formic acid in 10 mmol·L^−1^ ammonium formate water and (B) acetonitrile. The mobile phase was programmed as follows: an isocratic elution of 55% eluent B for 25 min. The flow rate was 0.6 mL·min^−1^ and the detection wavelength and the column temperature were set at 262 nm and 30 °C, respectively.

For the analysis of tetrahydropalmatine, dauricine, tetrandrine, homoharringtonine, harringtonine, and galanthamine (Table 2), the mobile phase consisted of (A) 0.2% formic acid in water and (B) acetonitrile. The gradient elution used for separation was programmed as follows: 5–10% eluent B from 0 to 5 min, 10–50% eluent B from 5 to 15 min, and 50–90% eluent B from 15 to 25 min. The flow rate was 0.8 mL·min^−1^; the column temperature was set at 30 °C. The detection wavelengths of dauricine, tetrandrine, homoharringtonine, harringtonine, and galanthamine were set at 280 nm, 282 nm, 282 nm, 288 nm, 288 nm, and 228 nm, respectively.

IT-TOF mass spectrometer was connected to an HPLC system via an ESI interface. Both positive and negative ESI were used to analyze the metabolites, and the parameters were as follows: CDL temperature, 200 °C; heat block temperature, 200 °C; detector voltage, 1.75 kV; nebulizing gas flow rate, 1.5 L·min^−1^; and drying gas pressure, 120 kPa. The ion accumulation time and precursor ion isolation width were set at 20 ms and 3 Da, respectively. Mass spectra were acquired in the range of *m*/*z* 100–800 for MS^1^. The MS^n^ data were collected in automatic mode. The collision-induced dissociation (CID) energy was set at 50%.

Mass calibration was carried out prior to data acquisition using direct infusion of a reference standard from 200 to 2100 Da. The flow rate of the infusion pump was 5 mL·min^−1^. The standard sample consisted of 0.25 mL/L trifluoroacetic acid and 0.1 g/L sodium hydrate. All calculated mass errors were less than 5ppm after mass calibration with the reference standard. The LC/MS solution version 3.60.361 software supplied with the instrument was used to carry out data acquisition and processing. An accuracy error threshold of 20 ppm was set as a limit to the calculation of possible elemental compositions.

### 3.6. Molecular Docking Between Eight Isoquinoline Alkaloids and 14α-Demethylase

Discovery Studio Client software (v16.1.0.15350) was used to compute the possible interaction of eight isoquinoline alkaloids on 14α-demethylase, whose crystal structures are available in the Protein Data Bank [22]. We used CDOCKER in the analysis of the binding between hit compounds and the protein. Except for pose cluster radius being set at 0.5, the docking parameters were set to the default values. The binding modes of compounds to the enzyme were chosen to further optimize the docking conformation according to their binding free energy.

## 4. Conclusions 

In this study, an accurate, selective, and sensitive method, LC/MS^n^-IT-TOF was developed to determine the metabolites of eight isoquinoline alkaloids; this method was successfully used for online analysis of the in vitro metabolites in the intestinal flora in our study. Four isoquinoline alkaloids containing a nitro-hexatomic ring (palmatine, tetrahydropalmatine, dauricine, and tetrandrine) were easily transformed by the intestinal flora in vitro, and a total of 9 metabolites were detected and characterized. The main metabolic pathway was the demethylation reaction. The other four isoquinoline alkaloids (sinomenine, homoharringtonine, harringtonine, and galanthamine), which contain a benzazepine structure, could not be transformed to demethylated metabolites by intestinal microbes in vitro. We also performed a computer-assisted docking of the initial interaction between the isoquinoline alkaloids and CYP51. The computational analysis that validated by the in vitro experiments determined that: first, the hydrophobic interactions were the main binding interactions between the ligands and active site residues; and second, the steric hindrance effect created by the benzazepine structure led to a resistance that resulted in a weak interaction between the compounds and the active site. Thus, it was suggested that there might be CYP51 or other metabolic enzymes with similar functions existing in the gut microbiota. In conclusion, our results demonstrated the importance of further fundamental work to fully exploit the biotransformation of isoquinoline alkaloids in vivo. This research will expand our understanding of the important role of the intestinal microbiome in drug metabolism and investigation. 

## Figures and Tables

**Table 1:** LC/MS^n^-IT-TOF data obtained for palmatine, tetrahydropalmatine, dauricine, tetrandrine and their metabolites from incubation with intestinal flora in vitro.

	t_R_(min)	MS^1^[M + H]^+^	MS^1^[M + 2H]^2+^	Fragments	
				MS^2^ *m*/*z*	MS^3^ *m*/*z*
Palmatine	10.9	352.1549	--	308.1316, 320.1315 291.1274, 337.1345 336.1270, 307.1144	262.0899, 246.0928 234.0912, 277.0773 290.0856, 217.0861 249.0785, 292.0991 264.1043
M1	9.2	338.1932	--	323.1150, 294.1120 339.1400, 262.0863 279.0867, 308.0943	279.0880
M2	9.2	338.1932	--	323.1150, 294.1120 339.1400, 262.0863 279.0867, 308.0943	279.0880
M3	9.5	324.1249	--	--	--
M4	8.2	310.1154	--	295.0828, 267.0837	297.0936, 266.0826 251.0483, 267.0916
Tetrahydropalmatine	13.5	356.1867	--	192.0936, 308.1205 340.1479, 165.0855 150.0612, 176.0643 338.1975, 204.0948	177.0717, 176.0655 165.0817, 148.0697 131.0665, 193.0963 150.0631, 159.0646
M5	12.6	342.1622	--	192.1041, 165.0932 150.0689, 310.1417 178.0886, 210.1703 135.0425	177.0767, 148.0744 159.0649, 131.0724 165.0937
M6	11.8	328.1471	--	164.0707, 192.1020 150.0677, 137.0582 280.1034, 312.1236	137.0616, 150.0686 165.0807, 119.0500
Dauricine	11.9	--	313.1673 (2)	313.1699 (2), 297.6498 (2) 282.1284 (2), 266.6156 (2) 290.1368 (2), 406.2091 194.0843	313.6692 (2), 297.6483 (2) 290.1350 (2), 282.1252 (2) 209.6039 (2), 266.6156 165.0931
M7	11.4	--	306.1574 (2)	290.6376 (2), 275.1159 (2) 297.6397, 284.6355 269.6195, 259.0936 290.6425, 282.1292	275.0978
M8-1	12.6	--	299.1083 (2)	283.6370, 290.6376 277.6312, 268.1075 213.0712	268.1075
M8-2	14.8	--	299.1083 (2)	283.6370, 290.6376 277.6312, 268.1075 213.0712	268.1075
Tetrandrine	12.9	--	312.1678 (2)	296.6400 (2), 281.1251 (2) 198.1021 (2), 366.1744 456.2231, 175.0939 312.6609, 122.0746 231.0251	297.1343 (2), 198.0982 (2) 236.0983, 158.0721 122.0712, 274.1006 188.0855
M9-1	10.5	--	305.1561 (2)	191.0947, 190.0876 288.6181, 281.1111 228.5973, 182.5820 367.1695	167.0633, 177.0823 168.0735
M9-2	12.0	--	305.1561 (2)	191.0947, 190.0876 288.6181, 281.1111 228.5973, 182.5820 367.1695	167.0633, 177.0823 168.0735

**Table 2:** HPLC analytical conditions of eight isoquinoline alkaloids.

Compounds	Time (min)	Mobile Phase		Flow Rate (mL·min^−1^)	Detection Wavelength (nm)	Column Temperature (°C)
		A	B			
Palmatine		0.5% formic acid in water	acetonitrile	0.8	347	40
	0	85%	15%			
	10	55%	45%			
	15	40%	60%			
	18	80%	20%			
	25	85%	15%			
sinomenine		0.1% formic acid in 10 mmol·L^−1^ ammonium formate water	acetonitrile	0.6	262	30
	0	45%	55%			
	25	45%	55%			
tetrahydropalmatine		0.2% formic acid in water	acetonitrile	0.8	280	30
	0	95%	5%			
dauricine	5	90%	10%		282	
tetrandrine	15	50%	50%		282	
homoharringtonine	25	10%	90%		288	
harringtonine					288	
galanthamine					228	
